# The Challenge of Long COVID-19 Management: From Disease Molecular Hallmarks to the Proposal of Exercise as Therapy

**DOI:** 10.3390/ijms232012311

**Published:** 2022-10-14

**Authors:** Raffaele Scurati, Nadia Papini, Paola Giussani, Giampietro Alberti, Cristina Tringali

**Affiliations:** 1 Department of Biomedical Sciences for Health, Università degli Studi di Milano, 20133 Milano, Italy; 2 Department of Medical Biotechnology and Translational Medicine, Università degli Studi di Milano, LITA Segrate, 20090 Segrate, MI, Italy

**Keywords:** long COVID-19, exercise, inflammation, health

## Abstract

Long coronavirus disease 19 (COVID-19) is the designation given to a novel syndrome that develops within a few months after infection by severe acute respiratory syndrome coronavirus-2 (SARS-CoV-2) and that is presenting with increasing incidence because of the numerous cases of infection. Long COVID-19 is characterized by a sequela of clinical symptoms that concern different organs and tissues, from nervous, respiratory, gastrointestinal, and renal systems to skeletal muscle and cardiovascular apparatus. The main common molecular cause for all long COVID-19 facets appears to be related to immune dysregulations, the persistence of inflammatory status, epigenetic modifications, and alterations of neurotrophin release. The prevention and management of long COVID-19 are still inappropriate because many aspects need further clarification. Exercise is known to exert a deep action on molecular dysfunctions elicited by long COVID-19 depending on training intensity, duration, and continuity. Evidence suggests that it could improve the quality of life of long COVID-19 patients. This review explores the main clinical features and the known molecular mechanisms underlying long COVID-19 in the perspective of considering exercise as a co-medication in long COVID-19 management.

## 1. Introduction

The first official appearance of the coronavirus disease 2019 (COVID-19) has been dated to the end of December 2019, when atypical cases of pneumonia were identified in Wuhan, China. Rapidly, the disease concerned all countries of the world, giving rise to the worst pandemic of this millennium [1]. The etiologic agent of COVID-19 has been recognized as the severe acute respiratory syndrome coronavirus (SARS-CoV)-2, belonging to the genus *Betacoronavirus* [2]. SARS-CoV-2 enters into cells mainly through the angiotensin-converting enzyme 2 (ACE2) receptor that was recognized, several years before, as the target of SARS-CoV [3]. The virus–receptor binding is mediated by interaction with the spike (S) glycoprotein, which is present, in many copies, as a homotrimer protruding from the viral surface and giving rise to the archetypal crown facet [4]. It has been strongly hypothesized that SARS-CoV-2 could enter into cells by also employing other receptors, such as C-type lectins, CD209 [5]. Nevertheless, the profile of expression of ACE2 appears to be the main factor determining the clinical appearance of COVID-19. ACE2 is expressed by a wide-ranging repertoire of cells, including nasal ciliated and upper bronchial cells, type II alveolar cells in lungs, small intestine and colon enterocytes, gallbladder, testis, kidney, thyroid, heart, and vascular cells [6]. Thus, it is possible to explain the different symptomatology of COVID-19 that, even if it manifests itself primarily as a respiratory disease, also affects other organs [7].

Currently, the pandemic is slowly succeeding into an endemic phase, mainly through the broad employment of vaccination and the production of novel antiviral drugs [8]. Nevertheless, the ability of SARS-CoV-2 to continuously generate different variants able to escape immunity acquired through vaccination or previous infections is deeply stretching the control of novel infections and re-infections. Moreover, a new challenge is being increasingly shown along with the increased number of infections. A consistent percentage of patients affected by COVID-19, often unrelatedly to the severity of the acute disease, continue to present a sequela of difficult-to-treat different clinical symptoms also for several months after the resolution of the acute infection. This novel illness, known as post-COVID-19 condition or long COVID-19, was defined by the World Health Organization (WHO) as a syndrome hitting people subjected to a previous SARS-CoV-2 infection and that occurs within three months from acute COVID-19 and lasts for about two months. Symptoms should not be referred to as other possible causes [9]. It is quite difficult to establish the incidence of long COVID-19 due to the heterogeneity of studies that explored this cue. Overall, it has been estimated that about 10% to 35% of recovered subjects are affected by long COVID-19, with a higher incidence recorded in hospitalized patients [10]. Indeed, long COVID-19 has been widely recognized as an emerging clinical issue also in children and adolescents previously affected by the mild or asymptomatic disease. A recent study investigating the outcome in Danish children affected by COVID-19 demonstrated the presence of long-term symptoms in children aged 0–14 years [11].

Long COVID-19 can show a remitting–relapsing trend, involves multiple organs, and can deeply impair the lifestyle. The factors that increase the risk of developing long COVID-19 are still not fully understood. They could differ from those causing a higher risk of developing a severe form of acute COVID-19. In a study, risk predictors of developing long COVID-19 were identified as age > 40 years, female gender, hospitalization, and frailty [12]. In children, older age and the pre-existence of allergies were identified as risk factors [13]. It must be emphasized that these data are constantly evolving; it could be crucial to identify which patients could be more at risk and, therefore, to begin early preventive treatment. Unfortunately, no specific pharmacological treatments could be addressed to prevent or treat long COVID-19 but symptoms are individually cured [10].

The comprehension of the molecular basis of long COVID-19 identifies physical activity as a strategy that could effectively prevent or ameliorate the syndrome. These findings strongly suggest the need for connecting molecular investigation of long COVID-19 and health research with exercise knowledge. With the appropriate validation, exercise prescription could become a key part of long COVID-19 prevention and treatment, following the canons of the initiative promoted by the American College of Sports Medicine (ACSM) in 2007 “The Exercise is Medicine” [14].

On these premises, the aims of this review are: (a) to discuss the clinical features of long COVID-19 along with their molecular basis; (b) to discuss why and how exercise could be effective in modifying the molecular alterations responsible for long COVID-19.

## 2. Clinical Symptoms and Laboratory Findings of Long COVID-19

**Figure 1:**
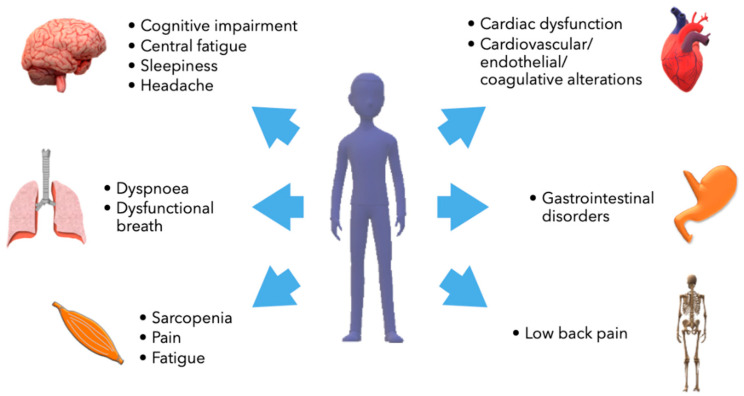
Main organs involved in long COVID-19.

Long COVID-19 looks like a multisystem syndrome affecting respiratory, neurological/cognitive, cardiovascular, endocrine, gastrointestinal, and musculoskeletal performance (Figure 1). Characteristic symptoms are fatigue and chronic weakness, cognitive impairment and difficulty concentrating, sleep disorders, headache, and cardiac alterations [15]. In some cases, symptoms are directly related to the organ damage elicited by the acute phase of the infection. Instead, organic alterations have not been recognized in patients affected by mild or pauci-symptomatic acute disease [16]. Most patients affected by long COVID-19 do not present significant alterations during a physical examination. Occasionally, weight loss could be revealed. Increased heart rate after standing has been described [10]. Glycemic alterations were identified for at least two months after the resolution of the acute infection [17]. Other significant abnormalities identified through biochemical laboratory testing are increased levels of ferritin, C-reactive protein, and D-dimer, reduced concentrations of hemoglobin and albumin, raised erythrocyte sedimentation rate (ESR), and, less frequently, boosted levels of serum lactate dehydrogenase [18].

### 2.1. Long COVID-19 and Neurological Impairment

Around one-third of patients showed signs of neurological and psychiatric disorders during the first six months after acute COVID-19 [19]. Cognitive impairment has been widely documented to occur in both hospitalized and never hospitalized COVID-19 patients [20,21]. Typical signs of this condition are difficulties in attention, short-term memory, and a sort of confused status referred to as “brain fog”, mood disturbance, dizziness, hyposmia, hypogeusia, tinnitus, tremors, hypoesthesia, and the onset of psychiatric syndromes and psychoses. Also, neuropathies, dementia, and cerebrovascular disorders have been described [21,22,23]. In a study performed on adolescents previously infected by SARS-CoV-2, the circulating levels of nerve growth factor (NGF) and brain-derived neurotrophic factor (BDNF) were measured 30–35 days after the last molecular test. NGF levels were lower than in controls, whereas BDNF was higher only in girls who had developed an acute symptomatic form of the disease [24]. Hypometabolic areas (in the rectal/orbital gyrus, olfactory gyrus, amygdala, hippocampus, hypothalamus, brainstem, thalamus, and cerebellum) were detected using brain positron emission tomography (PET) in long COVID-19 patients [25]. Neuroinflammation with alterations of microglia functionality has been suggested to be involved [26].

### 2.2. Long COVID-19 and Respiratory Impairment

Dyspnea and shortness of breath are common in long COVID-19 [27]. These symptoms do not seem to be related to organ damage in patients affected by a mild form of COVID-19; instead, they are the consequence of respiratory organ dysfunctions, such as lung fibrosis, in previously hospitalized patients. Dyspnea induced by exercise is related to hyperventilation that can arise from an insufficient energetic supply and autonomic dysfunction [28]. However, in a different study, cardiopulmonary exercise testing was performed in patients aged 64 years affected by long COVID-19 and persistent dyspnea. The test revealed dysfunctional breaths but devoiced hyperventilation [29]. Respiratory muscle dysfunction was identified in long COVID-19 patients [29].

### 2.3. Long COVID-19 and Cardiovascular Impairment

Severe acute COVID-19 is frequently associated with cardiovascular disorders, particularly thromboembolic events and cardiac alterations that could be fatal [30]. After recovery, cardiovascular abnormalities can persist. Conduction abnormalities, palpitation, left ventricle mechanical desynchrony, and above all postural orthostatic tachycardia syndrome are frequently described in long COVID-19 [31,32,33]. Endothelial dysfunctions were identified for several months after COVID-19 [34]. Studies based on magnetic resonance imaging proved that 78% of patients presented myocardial alterations and 60% myocarditis 2–3 months after COVID-19. Others demonstrated that 37% of patients with previous infections that occurred 10 weeks earlier also without symptoms, developed myocarditis [35,36]. However, a recent study reported that no cardiac abnormalities were identified in patients with self-reported symptoms that theoretically could be associated with cardiac dysfunction, such as dyspnea, chest pain, and palpitation [37].

It has been suggested that COVID-19-related-myocarditis could occur in young athletes with an estimated prevalence ranging from 1 to 4% [38].

Patients who recovered from COVID-19 have been demonstrated to be at high risk of thromboembolism. Many of them continue to present high levels of serum ferritin, D-dimer, factor VIII, and plasminogen activator inhibitor-1 (PAI-1) [18,39,40]. Current data support the notion that abnormal activation of the coagulation cascade persists after acute COVID-19. In addition, it was demonstrated that sera of long COVID-19 patients are characterized by a significant number of amyloid aggregates, mainly containing serum amyloid A and α2-antiplasmin and resistant to fibrinolysis [41,42].

### 2.4. Long COVID-19 and Musculoskeletal Impairment

SARS-CoV-2 affects, directly and indirectly, skeletal muscles, causing weakness, myalgia, and atrophy [43]. These symptoms often persist after recovery. Indeed, the most prevalent muscle manifestations of long COVID-19 are myalgia, muscle weakness, decreased muscular strength and a decline in physical performance, which affects the ability to perform daily activities and quality of life [44,45]. Muscle dysfunction may give rise to muscle atrophy characterized by a decrease in muscle mass, a decrease in capillarization, and mitochondrial impairment [44]. In patients hospitalized and treated in intensive unit care (ICU) during the acute disease, it is important to consider that immobility, muscle disuse, and pharmacological agents (glucocorticoids, chloroquine and hydroxychloroquine) contribute to skeletal muscle impairment causing significant reductions in skeletal muscle mass and strength and a high prevalence of sarcopenia. The fatigue associated with long COVID-19 results from central factors (such as neuroinflammation) but also from persistent inflammation in muscle fibers and neuromuscular junctions, sarcolemma damage, fiber necrosis, and skeletal muscle atrophy [15]. Recently a web-based study demonstrated the occurrence of fibromyalgia in patients with long COVID-19, with an estimated prevalence of over 30% [46]. The Authors defined this manifestation as “FibroCOVID”. Unfortunately, the survey did not accurately consider the relationships between the clinical severity of acute COVID-19 and the development of fibromyalgia but identified male gender and obesity as independent and strong risk factors for post-COVID fibromyalgia [46]. Muscular manifestations such as rhabdomyolysis and autoimmune myositis are less frequent but reported in several patients [44,47,48].

Rheumatic and skeletal pains are quite frequent during the acute phase of COVID-19 and sometimes persist for months. A study demonstrated that, three months after the hospitalization, 74.6% of patients presented at least one of these symptoms [49]. After six months, joint and back pain continued to be observed in a significant percentage of those healed from COVID-19 whereas many patients reported new-onset pain [50].

## 3. The Molecular Hallmarks and Dysfunctions of Long COVID-19

The pathogenesis of long COVID-19 is still poorly known, above all in patients who recovered from a mild acute form without any apparent organ damage resulting from the acute infection. It appears plausible that most of the symptomatology of this heterogeneous syndrome could be attributed to the persistence of inflammatory and autoimmune status. At this moment, it is not certain if this condition could be induced by the remaining presence of SARS-CoV-2 in local districts, although it is no longer detectable in nasal-pharyngeal swabs [51]. To support this last hypothesis several papers identified the persistence of SARS-CoV-2 in fecal specimens of patients with negativized pharyngeal swabs [52] and its nucleic acids in intestinal biopsies taken from half of the patients previously infected after more than six months [53]. Other possibilities that are under investigation are linked to autoimmunity triggered by antigenic cross-reactivity or to organ damage repair [54]. SARS-CoV-2, unlike other coronaviruses, elicits a persistent inflammatory status also after a mild acute disease. Acute COVID-19 includes many pathological aspects determined by inflammation, such as the strong increase in cytokines, the so-called “cytokine storm” related to worsening a patient’s health [55]. Autopsies of deceased COVID-19 patients have demonstrated that organ failure is mostly due to an over-activated immune response [56].

Indeed, many pieces of evidence concur in proving that the long-term effects of SARS-CoV-2 infection could be caused by persistent dysfunctions of the immune response, with chronic activation of T and B lymphocytes [26]. In previously hospitalized patients, T cell activation markers (CD69, OX40, CD154, and Human Leukocyte Antigen-DR isotype (HLA-DR)) were found to be over-expressed, whereas T cell exhaustion markers, i.e., programmed death-ligand 1 (PD-L1), and T cell immunoreceptor with immunoglobulin and ITIM domain (TIGIT), were found to be elevated in both hospitalized and non-hospitalized patients. Adaptive and innate immune cells were found to be decreased in hospitalized patients, even if B lymphocytes increased. These immune alterations were demonstrated to persist for a long time after SARS-CoV-2 infection [57]. Similarly, an inflammatory immune signature in the early recovery stage after COVID-19 was demonstrated by Wen et al., along with the decrease in circulating CD4^+^ and CD8^+^ T lymphocytes and the increase in CD14^+^ monocytes expressing inflammatory genes. Naïve B cells were reduced instead of plasma B cells which increased [58]. Lymphopenia and other immune alterations were confirmed by another work in subjects analyzed at 12- and 16-weeks post-infection. CD3^+^ and CD19^+^ T cells and CD19^+^ and CD38^+^ CD27^+^ memory B cells were found to be increased, whereas CD4^+^ and CD8^+^ T cells decreased. Natural killer (NK) cells and granulocytes increased [59]. A deep investigation of T cell subtypes identified the decrease in Th9 CD4^+^ T cells that were hypothesized to migrate to sites of inflammation, and the increase in Th2/22 CD4^+^ T cells. Further, naïve T regulatory (Treg) cells increased during the post-infection period, whereas central memory (CM) and effector memory (EM) Treg cells decreased [59]. Treg cells are specifically involved in modulating/suppressing the immune response, thus avoiding the onset of autoimmunity. Among them, EM Treg cells can induce an immediate and strong suppression, CM Treg cells are less suppressive, and naïve Treg cells are even less suppressive but highly proliferative [60]. Thus, it seems that during COVID-19 recovery an impairment of Treg differentiation occurs which could expose the risk of autoimmune or immune disorders. Several months after the infection, several cytokines, such as type I and III interferons (IFNs), remain higher and persistent effects on innate and adaptive immune effectors were determined [54].

Immune dysfunctions have been related to the impairment of the hypothalamic–pituitary–adrenal gland (HPA) axis and adrenal gland insufficiency resulting in hypocortisolemia [61]. Cortisol is known to be a stress hormone, also involved in inflammation, glycaemia, and sleep control. Thus, hypocortisolemia could be related to the difficulty in reducing the inflammatory response. Also, the Epstein virus and herpesviruses reactivation has been associated with low cortisol levels [62]. The causes underlying hypocortisolemia could be multiple and are under investigation: direct infection of the adrenal gland, glucocorticoid receptor alpha tissue resistance, the onset of secondary adrenal gland insufficiency due to glucocorticoid therapy, and the appearance of anti-adrenocorticotropic hormone (ACTH) antibodies have been reported [63]. Low levels of cortisol associated with altered HPA axis have also been described in other clinical conditions such as chronic fatigue syndrome and myalgic encephalomyelitis/fibromyalgia. Thus, hypocortisolemia could arise from HPA axis insufficiency after long stressful periods and the entity of this alteration could help to identify patients at risk of developing long COVID-19 [61].

Dysfunctions in the innate immune response, in particular related to the interferon pathways, could also arise from the hijacking of mitochondria induced by SARS-CoV-2. This event primarily increases the formation of double-membrane vesicles involved in virus replication and ultimately leads to the release of mitochondrial DNA, cytochrome C, and cardiolipin in the cytosol and mitophagy inhibition, promoting inflammation and multiple organ damage [64] or cognitive impairment [65]. Mitochondrial damage induced by SARS-CoV-2 can exacerbate pre-existing mitochondrial alterations typical of ageing, obesity, and other chronic conditions [64].

Autoantibodies have been supposed to play a role in long COVID-19, and persisting autoimmunity has been demonstrated [66]. Anti-G protein-coupled receptors (GPCR) autoantibodies have been identified in long COVID-19 patients; they are commonly associated with cardiovascular, nervous, and pulmonary manifestations [56,67]. Also, autoantibodies toward the skin, skeletal muscle, and cardiac targets, antinuclear and antineutrophil cytoplasmic antibodies have been detected [68,69]. It was hypothesized that post-COVID-19 autoimmunity is caused by a loss of self-tolerance and subsequent altered immune activation in genetically susceptible subjects [70]. This hypothesis is supported by the evidence that SARS-CoV-2 induces a severe inflammatory response, lymphopenia, complement consumption and autoantibodies formation only in some patients. When the infection is under control, lymphocyte count normalizes but some alterations persist [70]. Autoimmunity has also been related to molecular mimicry between peptides belonging to SARS-CoV-2 S glycoprotein and some human antigens particularly expressed by endocrine glands or cells such as the pituitary, adrenal glands [71], thyroid, and pancreatic beta-cells [72]. Recently, a characteristic immunologic profile characterized by low levels of neutralizing and anti-S antibodies with high anti-nuclear autoantibodies (ANA) and inflammatory markers was detected in patients reporting long COVID-19 symptoms for 1-year, demonstrating the presence of long-term immune system perturbations and autoimmunity [73].

The establishment of this chronic low pro-inflammatory status has been related to endothelial and vascular alterations. In recovered patients, high levels of circulating endothelial cells, which are released from blood vessels after vascular damage, were measured. Moreover, activated markers recognized by CD8^+^ T cells were identified on circulating endothelial cells and it was hypothesized that a cytotoxic immune response toward endothelium occurs [74]. Endothelium activation represents a significant risk of developing cardiovascular diseases for several months following infection.

Many patients affected by long COVID-19 present symptoms of orthostatic intolerance. Orthostatic intolerance occurs during standing up. In fact, during this action, thoracic blood is rapidly diverted downward, and circulatory and neurologic responses are required to maintain blood pressure. In particular, heart and aortic baroreceptors are stimulated and, in turn, increase sympathetic neural and adrenergic tone, inducing the release of noradrenaline and adrenaline. Adrenaline and noradrenaline release induces strong tachycardia, breathlessness, altered vision, exercise intolerance, weakness, and so on. It has been supposed that the release of pro-inflammatory cytokines and autoantibodies, the altered immune response, or SARS-CoV-2 itself could alter the autonomic nervous system [75]. Noticeably, autoimmunity has already been associated with orthostatic intolerance in the past [76,77].

Investigating the molecular alterations displayed by patients affected by long COVID-19, demonstrated that a significant telomere shortening occurs in parallel to a decrease in ACE2 gene expression. Thus, it was supposed that epigenetic modifications could be related to post-COVID-19 [78]. Further, it was suggested that COVID-19 could induce epigenetic ageing which, in turn, could contribute to long COVID-19 [79]. Long COVID-19 was also associated with alterations in the gut microbiome that could be related to persistent respiratory and neuropsychiatric symptoms, and fatigue [80,81,82]. Given the role of the gut microbiome in regulating epigenetics [83], it could be speculated that its modifications are also involved in long COVID-19 effects on epigenetics.

**Figure 2:**
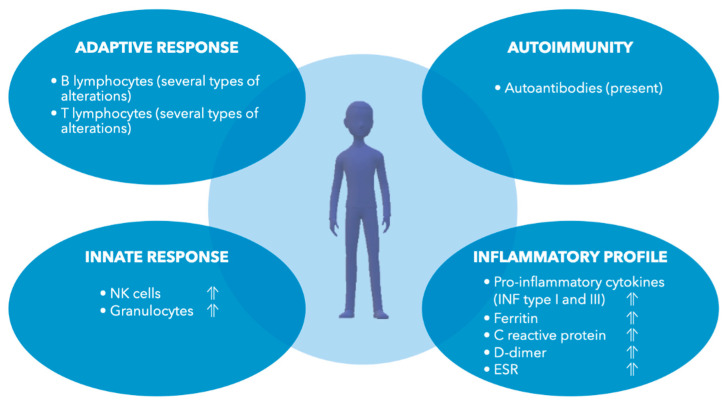
Immunological and inflammatory alterations described in long COVID-19.

Thus, summing up, it seems that the molecular mechanisms underlying long COVID-19 mostly consist of immune dysfunctions leading to multiple subsequent effects (Figure 2). Epigenetic and other alterations of neurotrophin levels complete the disease’s molecular signature.

## 4. Why Physical Activity Could Modulate the Molecular Phenotype of Long COVID-19

To understand if physical activity could be a realistic tool to treat long COVID-19, it is important to define how it can influence the causative mechanisms of this syndrome. Indeed, exercise modulates many molecular pathways that seem to be related to the pathogenesis of long COVID-19.

Firstly, regular exercise greatly impacts the regulation of the immune response. It is well-known that people habitually performing moderate exercise are less affected by mild infections such as upper respiratory tract infections than sedentary people, whereas intense exercise induces transient immunosuppression [84]. In detail, exercise has deep effects on the mobilization and activation of innate immune cells, including neutrophils, inflammatory monocytes, dendritic, and NK cells [85]. Regarding the effects of exercise on adaptive immune response, training, intensity, and duration have been demonstrated to modify the release of salivary immunoglobulin (Ig) A and increase the number of circulating B cells [85]. Also, the T cell number is modulated accordingly to exercise intensity and duration; generally, T lymphocytosis is observed immediately after an intense exercise, and then a decrease in circulating T cells is recorded for about 3 h [85]. Regular exercise leads to persistent adaptations that consist of immune suppression in the case of intense training and, on the contrary, immune boosting in the case of moderate training. Further, acute and chronic exercise regulates Treg cell phenotype and distribution [86].

Secondly, exercise exerts both acute and long-term anti-inflammatory effects, demonstrated by the decrease in inflammatory markers, such as C-reactive protein, through different routes. During or immediately after the exercise, to ensure energy supply, muscle contraction induces interleukin (IL)-6 synthesis, in a tumor necrosis factor (TNF)-α independent manner, and IL-6 release by the muscle itself. IL-6 increase does not consist of a pro-inflammatory response during this phase. In fact, muscle-resident pro-inflammatory 1 (M1) macrophages decrease, whereas anti-inflammatory 2 (M2) macrophages increase [87]. In addition, IL-6 promotes the release of anti-inflammatory cytokines such as IL-1receptor antagonist (Ra) and IL-10, decreasing at the same time that of the pro-inflammatory TNF-α [88]. Thus, moderate exercise is associated with an immediate anti-inflammatory acute response. Instead, long-term anti-inflammatory effects of exercise appear to be mostly mediated by the action on Treg cells [86] and by the reduction in adipose tissue and, as a consequence, in the release of IL-6 and TNF-α from macrophages localized in this tissue [87]. Moreover, endurance training, such as running training, promotes a fiber-type switching toward oxidative type I, a process that is accompanied by an increase in the expression of the peroxisome proliferator-activated receptor-gamma coactivator-1α (PGC-1α). PGC-1α stimulates mitochondria biogenesis, improves oxidative metabolism, and reduces local inflammation, decreasing oxidative stress, IL-6 and TNF-α levels, and inducing the polarization from M1 to M2 muscle macrophages [87]. In murine models of acute lung injury, it was demonstrated that moderate exercise reduces neutrophil recruitment in injured lungs and alveolitis. This event is mediated by a reduced release of IL-17F and granulocyte colony-stimulating factor (G-CSF) and decreases lung inflammation, and ameliorates respiratory distress [89]. Moreover, exercise produced an anti-inflammatory effect in women affected by multiple sclerosis, reducing the release of IL-17 and INF-γ [90]. However, it should be underlined that exhaustive exercise could induce the opposite effects increasing IL-6, IL-17, and IL-23 release and neutrophil activation [91].

For all these reasons, regular, moderate exercise is now recommended to alleviate symptoms of chronic inflammatory rheumatic and musculoskeletal diseases [87] and immune diseases such as multiple sclerosis [92], and in all other diseases characterized by low-grade systemic inflammation [93].

Importantly, exercise dosage in terms of type, intensity, frequency, and duration are closely related to anti-inflammatory and well-being effects [94]. Thus, a correct prescription of physical activity could be planned to dampen inflammation.

In addition, other effects mediated by exercise could be relevant for the treatment of long COVID-19. Exercise is crucial to promote muscle mass and therefore counteract sarcopenia. Both endurance and resistance training reduce myostatin levels. Moreover, the Akt-mammalian target of rapamycin (mTOR) pathway and downstream protein synthesis are particularly stimulated by resistance training such as weight training, whereas the muscle RING-finger protein-1 (MuRF-1) and Atrogin-1 are decreased [95]. It was demonstrated that patients with long COVID-19 present metabolic alterations such as impairment of fatty acid β-oxidation and increased lactate release during exercise, possibly associated with mitochondrial dysfunctions [96]. Acute exercise activates molecular pathways leading to mitochondria biogenesis and to the induction of cellular autophagy that accelerates mitochondria turnover itself. Repeated exercises increase mitochondria and improve electron transport chain [97]. Further, exercise has been demonstrated to be efficacious in reversing and strengthening impaired mitochondrial functionality in ageing or muscle disuse [97]. Moreover, exercise induces the release of nitric oxide in skeletal muscle, which activates AMP-activated protein kinase and PGC-1α, i.e., inducers of mitochondrial biogenesis [98]. Thus, to reduce fatigue, it could be relevant to understand if a correct exercise dosage could ameliorate mitochondria and metabolism dysfunctions and, as a consequence, the transition from anaerobic to aerobic/oxidative metabolism.

Exercise produces effects on the endothelium. In particular, enhanced laminar shear stress promoted by exercise stimulates signaling pathways downstream of the vascular endothelial growth factor (VEGF) receptor and platelet endothelial cell adhesion molecule 1 (PECAM1). Endothelial nitric oxide synthase (eNOS) is then activated by Akt-mediated phosphorylation. eNOS can also be activated by integrin downstream pathways [99]. Nitric oxide is largely involved in vascular and endothelial functionality because it promotes vasodilatation, modulates cell growth, and avoids platelet aggregation [100].

Finally, it has been largely recognized that acute exercise transiently ameliorates cognitive function, and that regular training activates neuro and synaptic plasticity and promotes neural survival. These effects are primarily induced by the exercise-mediated release of BDNF [101]. Thus, regular exercise results in a beneficial impact on memory, sleep, and mood, and for this reason, it is also recommended to prevent or treat neurodegenerative disorders [102].

Regular exercise may also induce qualitative and quantitative modifications in gut microbiota, improving immune responses, metabolic, and epigenetic functions [103].

**Figure 3:**
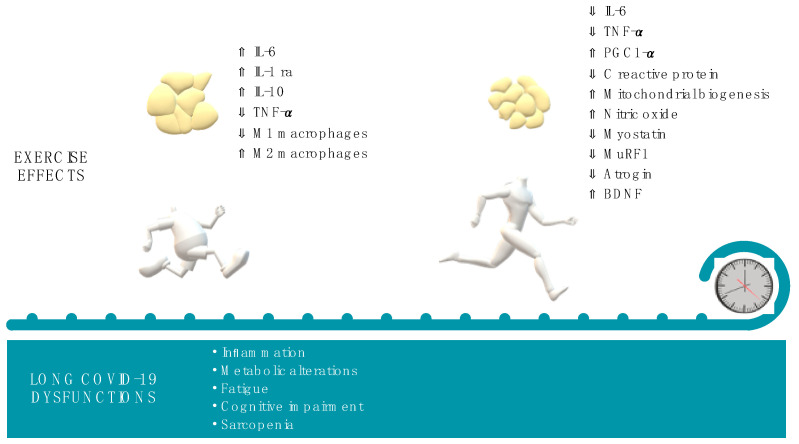
Effects induced by acute and chronic exercise on molecular mediators potentially involved in long COVID-19.

Summing up all these pieces of evidence, the correct dosage of exercise could be efficacious in reducing many aspects related to long COVID-19 (Figure 3).

## 5. Exercise Prescription as Medicine for Long COVID-19: Cues and Cautions

Rehabilitation operates in secondary (treatments during acute setting) and tertiary (post-syndrome) prevention and is aimed at designing interventions to reduce disability and post-syndrome risks [104]. Specifically, physical rehabilitation protocols represent one relevant asset during the post-COVID-19 recovery. A large consensus exists about the promotion of exercise to reduce symptomatic sequelae and restore and improve the quality of life of patients who recover after infection. Physical exercise has been generally recommended to address the consequences of the illness and the resulting forced inactivity, such as respiratory distress, physical deconditioning, and muscle weakness, and to support recuperating daily functioning [105]. In addition, the severity of post-COVID-19 syndrome has been supposed to reflect physical fitness and cardiopulmonary functionality, whose efficiency is often associated with low severity of symptoms. Therefore, a high fitness status at the onset of the disease would help limit the functional disability due to the acute infection [106], which suggests that physical exercise represents a primary prevention action.

Recent studies have addressed specific exercise protocols for rehabilitation after COVID-19, reporting generic final suggestions [107]. They commonly followed the American College of Sports Medicine FITT-VP (frequency, intensity, time, type, volume and progression) to approach exercise prescription [108] and considered: (i) when to begin exercising; (ii) frequency, intensity, and duration of the exercise; (iii) which type of activity should be performed; (iv) the assessments for participants’ evaluation and tailoring exercising. Rehabilitation programs should start within 30 days after the acute phase [109]. They must be tailored and individualized to improve the effectiveness of functional recovery [106]. According to the Stanford Hall Consensus Statement for post-COVID-19 rehabilitation [110], exercising over three metabolic equivalents of task (METs) should be avoided until two/three weeks after overcoming severe symptoms or one week after mild/moderate COVID-19. However, with very mild symptoms, only prolonged and exhaustive activity might be avoided, limiting activity to light efforts [110]: patients with mild symptoms can exercise, but they should not increase training during the acute phase of the illness [111]. Once recovered, a progressive increase in exercising is suggested and has been experimenting with various protocols lasting four weeks at least: 12 sessions (three 50-min/1-h sessions per week for four weeks, or two sessions per week for six weeks) [112,113], eight weeks twice a week [114], three times per week over eight weeks or longer [115], the activity’s intensity varying from medium to high. Cattadori et al. reported that the literature on COVID-19 exercising, by trials or expert consensus, suggests practicing aerobic and resistance training, respiratory, diaphragmatic and cough exercises, and stretching [107]. Physical, cognitive, and functional assessments are required to evaluate the actual state of patients, tailor the physical activity to administer, and better support patients in returning to their previous lifestyle and work [110,116].

Regarding long COVID-19 syndrome management and rehabilitation, the role of physical activity has not been fully clarified [117], and the grade of the effectiveness of exercise interventions is still under investigation [118]. However, there is no reason to undervalue exercise efficacy to preserve functionality or mitigate disability after the onset of the disease as positive effects against severe COVID-19 symptoms have been retrieved [106], suggesting further benefits to long COVID-19 manifestation. In addition, cardiopulmonary rehabilitation with moderate continuous aerobic training and resistance training has been proven beneficial for treating patients, increasing exercise tolerance, overcoming fatigue, and positively affecting psychological domains such as the perceived quality of life [119]. Adequate and tailored physical activity is therefore emerging as a co-treatment for alleviating long COVID-19 symptoms and accelerating recovery [120].

Defining appropriately tailored exercise is a pivotal issue in long COVID-19 exercise prescription and administration. Before planning an activity, the starting point should be setting the patients’ disability condition, e.g., by scales such as the Short Physical Performance Battery [107] and the Functional Disability Inventory [121], distinguishing between organ damage or functional deconditioning [122]. Even if the study was validated with a limited number of participants, Jandhyala et al. designed an instrument to classify subjects with long COVID-19 by observing some features of psychological, physical, social and work domains and measuring the impact of long COVID-19 on the quality of life. If it is confirmed, it would be beneficial to complete the baseline assessment before exercising [123]. Afterwards, the type of exercises to administer long COVID-19 patients and the tailoring criteria should be addressed; training loads, frequency, intensity, and duration should be appropriately set. Indeed, fatigue and neurocognitive symptoms are frequent and debilitating features impacting long COVID-19 syndrome patients [120] and reverberating to their quality of life. While suggested for rehabilitation, physical activity can intensify symptoms such as post-exertion malaise [117] or have mixed effects, which can improve or worsen symptoms [124]. For example, even if low loads, volumes, and not-to-failure repetitions in strength training can improve to some extent strength, power and muscle hypertrophy without recurring to maximal efforts and limiting typical discomfort and fatigue as traditional training [120], people with long COVID-19 syndrome often experience syndrome-related unwillingness in exercising and physical and psychological intolerance to physical activity. Rehabilitation should be restorative and not detrimental to daily activity: since, for long COVID-19 patients, moving in daily life might be considered physical activity, as well as autonomy and individuals’ perceptions and should be fundamental in any exercise intervention for such recipients [120], an exercise tailoring strategy such as pacing better counteract post-exertional malaises and is preferable than a grading strategy. Pacing is a self-managed use of physical and mental energy reserves, which prevents or mitigates symptom flare-ups [117] and can be applied to long COVID-19 by suggesting to stop trying to approach and reach their own limits, rest before symptoms of fatigue occur, and pace physical and cognitive activities [125]. Differently, graded exercise seems to worsen symptoms much more than pacing and, for this reason, in the UK, pacing has been recommended in treatments for those recovering from COVID-19 [124].

Cardiorespiratory fitness, muscle function, and joint health should be assessed to detect primary manifestations of long COVID-19 syndrome (such as exertional fatigue and dyspnea), tailor physical exercise, enhance functional capacity and health [108], and improve quality of life. Several papers reported that, among the functional screening and assessment procedures, the most popular seems to be the 6-Minute Walking Test to estimate the aerobic capacity and exercise tolerance [107,109,114,115,116,118,126] and the hand grip to measure strength [107,114,116]. In addition, laboratory tests such as spirometry and transthoracic echocardiogram are frequently used to evaluate cardiorespiratory functionality with direct measurements [107]. For this purpose, Jimeno-Almazan suggests also including physical fitness measures when assessing the health status of patients [106]. Dyspnea measurements can be accomplished by the British Medical Research Council Dyspnea Score [118]. Balance is a further common investigated feature, e.g., by the Berg Balance Scale for balance [108,116]. The measurement of fatigue, necessary to tailor or manage exercise execution and the consequent effects, is usually and regularly accomplished, generally by the 10- or 20-point Borg rating for perceived exertion scales [107,114,115,116,118,127,128]. Finally, behavioral psychology questionnaires such as the short form health survey 36 (SF-36) [115], the post-acute (long) COVID-19 quality of life [123], or the pediatric quality of life (PedsQL) inventory–parent proxy report [121] should be administered to evaluate the quality of life, which recovery or improvement is, in the end, the outcome of any therapy and rehabilitation intervention.

**Figure 4:**
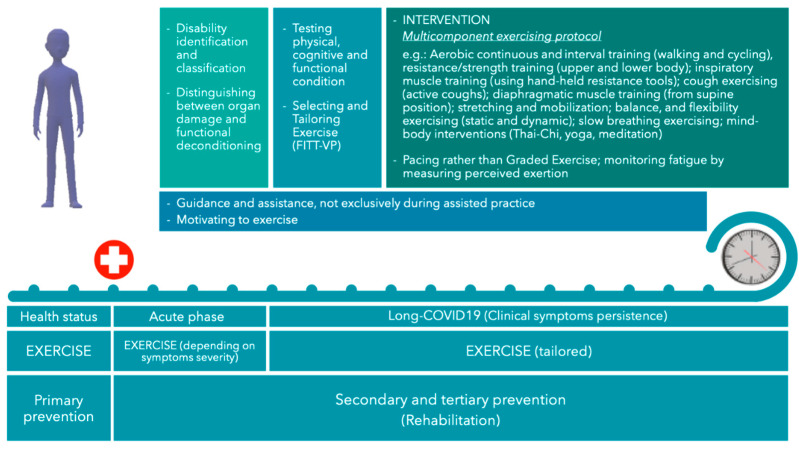
Exercise intervention with long COVID-19 patients. FITT-VP = frequency, intensity, time, type, volume, and progression.

Among the types of physical activity to be included in intervention programs, respiratory physiotherapy and aerobic and resistance training generally are helpful for dyspnea, fatigue and sarcopenia, which are the most frequent symptoms of the long COVID-19 syndrome [118]. Cattadori et al. concentrated on specific exercise guidelines they retrieved from original articles or review literature [107]. They suggested a multicomponent exercising protocol that includes aerobic continuous and interval training (walking and cycling), resistance/strength training (upper and lower body), inspiratory muscle training (using hand-held resistance tools), cough exercising (active coughs), diaphragmatic muscle training (from supine position), stretching and mobilization, balance, and flexibility exercising (static and dynamic), slow breathing exercising [107,113,114,115]. Among the more promising interventions, because of their physical (diaphragmatic breathing, postural alignment, cardiovascular, balance, and neuromuscular training) and psychological benefits (on cognitive functions, anxiety and depression), Tai Chi has been suggested as a multicomponent activity to counteract long COVID-19 [129]. Lastly, other than defining guidelines for planning and setting physical exercise for long COVID-19 patients, particular attention should be devoted to how to assist patients, not exclusively during assisted practice. Many people perceive a lack of care and support to counteract their long COVID-19 condition and feel frustrated and left self-managing exercising and establishing strategies for managing unassisted physical activities (e.g., daily activities such as housework and gardening). They often recur to online communities for advice and guidance [117]. Also, they often report poor information about how to address the physical and psychological consequences of the syndrome, which negatively impacts the sense of self and quality of life and causes discomfort with physical activity. Motivation to start exercising is challenging for people with chronic pain. The physical and mental approach is even more relevant with long COVID-19 patients and pleasant exercising, fun, and tolerated engagement must preserve overtraining by exceeding personal limits, preserving motivation and monitoring activity [120]. Figure 4 recaps a possible approach to the long COVID-19 occurrence.

## 6. Conclusions

Long COVID-19 is considered an emerging and worrying challenge related to the still present COVID-19 pandemic. Since 2019, different SARS-CoV-2 variants have developed, often with a higher transmission capability. However, it is unknown whether the possibility of long-term effects after the infection has also changed [16]. It can be hypothesized that the high rate of infections and re-infections recorded in this new phase of the pandemic could significantly increase the number of patients affected by long COVID-19. Unfortunately, long COVID-19 can significantly compromise life quality for many months. No consensus exists about managing this complex syndrome that causes the onset of symptoms associated with different tissues. For these reasons, it has been suggested that the assessment of multidisciplinary teams and a holistic approach to better address management of long COVID-19 [130]. There is no specific therapy, but only drugs targeting specific symptoms are prescribed without reaching overall significant wellness. As previously discussed, many papers indicated that physical rehabilitation and exercise could play an important role in the approach to long COVID-19, possibly the latter being valuable for both prevention and treatment [106]. It is important to underline that exercise is a unique strategy able to induce multiple positive effects on many organs simultaneously, contrary to drugs or other medications [14]. In this context, mind–body interventions such as Tai-Chi, yoga, and meditation could also integrate into the plan of training because of their important role in stress management and immune system modulation [131]. In light of current evidence, investigating molecular effects of exercise and protocols’ safety in long COVID-19 patients could be a promising perspective. Caution to employ training protocols not scientifically validated should be used: as for all medications, exercise may not be the correct solution for all patients. As previously discussed, screening based on physical capabilities and clinical features also considering organ failure or the presence of comorbidities should be performed before beginning any type of training. Based on previous experience with myalgic encephalomyelitis/chronic fatigue, a syndrome sharing many features with long COVID-19 and emerging after an infection in 80% of cases, exercise can be harmful if not correctly addressed [125]. The current knowledge of long COVID-19 does not allow stratifying patients into clusters that surely will benefit from exercise or have significant side effects. A better investigation of biomarkers modulated by exercise in long COVID-19 patients could be helpful to this end. Moreover, the continuous monitoring of patients during training and avoiding post-exertional malaise and overexertion exercises should be mandatory.

## Data Availability

Not applicable.
